# Appearance of Human Plasma Cells Following Differentiation of Human B Cells in NOD/SCID Mouse Spleen

**DOI:** 10.1080/10446670310001642122

**Published:** 2003

**Authors:** Kentaro Kikuchi, Zhe-Xiong Lian, Xiao-Song He, Aftab A. Ansari, Miyuki Ishibashi, Hiroshi Miyakawa, Leonard D. Shultz, Susumu Ikehara, M. Eric Gershwin

**Affiliations:** 1 Division of Rheumatology Allergy and Clinical Immunology University of California at Davis School of Medicine TB 192 Davis CA 95616 USA; 2 Department of Pathology Emory University School of Medicine Atlanta GA USA; 3 Fourth Department of Internal Medicine Teikyo University School of Medicine Kawasaki Kanagawa Japan; 4 Jackson Laboratory 600 Main Street Bar Harbor ME 04609 USA; 5 First Department of Pathology Kansai Medical University Moriguchi Osaka Japan

## Abstract

Relatively little is known for the differentiation and maturation process of human B cells to plasma cells. This is particularly important in reconstitution work involving transfer of autoantibodies. To address this issue, we transplanted human peripheral blood mononuclear cells (PBMC) directly into the spleen of irradiated NOD/SCID mice depleted of natural killer cell activity. Within 6 weeks, naïve B cells differentiated into memory B cells and, importantly, the numbers of human CD138^+^ plasma cells in spleen increased by 100 fold after transplantation. Plasma cell numbers correlated with the detection of human IgM and IgG in serum, indicating that human B cells had differentiated into mature plasma cells in the murine spleen. In addition to CD19^+^ plasma cells, a distinct CD19^-^ plasma cell population was detected, suggesting that downregulation of CD19 associated with maturation of plasma cells occurred. When purified human B cells were transplanted, those findings were not observed. Our results indicate that differentiation and maturation of human B cells and plasma cells can be investigated by transplantation of human PBMC into the spleen of NOD/SCID mice. The model will be useful for studying the differentiation of human B cells and generation of plasma cells.


*Clinical & Developmental Immunology*, June–December 2003 Vol. 10 (2–4), pp. 197–202

Taylor & Francis healthsciences

### Appearance of Human Plasma Cells Following Differentiation of Human B Cells in NOD/SCID Mouse Spleen

KENTARO KIKUCHI[a], ZHE-XIONG LIAN[a], XIAO-SONG HE[a], AFTAB A. ANSARI[b], MIYUKI ISHIBASHI[c], HIROSHI MIYAKAWA[c], LEONARD D. SHULTZ[d], SUSUMU IKEHARA[e] and M. ERIC GERSHWIN[a,]*

[a]*Division of Rheumatology, Allergy and Clinical Immunology, University of California at Davis School of Medicine, TB 192, Davis, CA 95616, USA;* [b]*Department of Pathology, Emory University School of Medicine,Atlanta, GA, USA;* [c]*Fourth Department of Internal Medicine, Teikyo University School of Medicine, Kawasaki, Kanagawa, Japan;* [d]*Jackson Laboratory, 600 Main Street, Bar Harbor, ME 04609, USA;* [e]*First Department of Pathology, Kansai Medical University, Moriguchi, Osaka, Japan*

Relatively little is known for the differentiation and maturation process of human B cells to plasma cells. This is particularly important in reconstitution work involving transfer of autoantibodies. To address this issue, we transplanted human peripheral blood mononuclear cells (PBMC) directly into the spleen of irradiated NOD/SCID mice depleted of natural killer cell activity. Within 6 weeks, naïve B cells differentiated into memory B cells and, importantly, the numbers of human $CD138^+$ plasma cells in spleen increased by 100 fold after transplantation. Plasma cell numbers correlated with the detection of human IgM and IgG in serum, indicating that human B cells had differentiated into mature plasma cells in the murine spleen. In addition to $CD19^+$ plasma cells, a distinct $CD19^-$ plasma cell population was detected, suggesting that downregulation of CD19 associated with maturation of plasma cells occurred. When purified human B cells were transplanted, those findings were not observed. Our results indicate that differentiation and maturation of human B cells and plasma cells can be investigated by transplantation of human PBMC into the spleen of NOD/SCID mice. The model will be useful for studying the differentiation of human B cells and generation of plasma cells.

*Keywords*: Autoantibodies; B cell ontogeny; Reconstitution; NOD/SCID mice

### INTRODUCTION

Plasma cells are terminally differentiated B cells that produce and secrete antibodies. Little is known about the development and differentiation of human B cells into plasma cells due to the low frequency of plasma cells in the peripheral blood. Murine models, especially immunodeficient $Prkdc^{scid}/Prkdc^{scid}$ (SCID) mice engrafted with human cells (SCID-Hu), have been used to investigate the development, differentiation and *in vivo* functions of human lymphocyte subsets (McCune *et al.*, 1988; Mosier *et al.*, 1988). Differentiation of human B cells have been examined by transplanting umbilical cord blood cells into the scid/scid mice (Mosier *et al.*, 1988; Pflumio *et al.*, 1996; Hogan *et al.*, 1997; Novelli *et al.*, 1999; Rossi *et al.*, 2001). However, when peripheral blood mononuclear cells (PBMC) were transplanted intraperitoneally, the majority of the identifiable human cell population were T cells (Mosier *et al.*, 1988). This has been a particular problem in attempts to transfer autoimmune disease using human PBMCs to SCID mice.

Naïve B cells, following generation in the bone marrow, migrate to secondary lymphoid organs before they differentiate into memory B cells and plasma cells under complex environmental stimuli (Calame, 2001). The spleen is known to be a major site of B cell differentiation. To explore the differentiation process of human B cells, we directly engrafted human PBMC into spleens of NOD/LtSz-scid/scid (NOD/SCID) mice.

### MATERIALS AND METHODS

### Engraftment of Human PBMC into NOD/SCID Mice

NOD/SCID mice, aged 4–5 weeks, were obtained from the Jackson Laboratory (Bar Harbor, ME, USA) and subsequently maintained by the Animal Resource Service of the University of California at Davis. Prior to engraftment, each mouse received 2 Gy of total body irradiation. Each mouse also received an intraperitoneal injection of an optimal dose of 20 μl of rabbit anti-mouse/rat asialo GM1 polyclonal antibody (Cedarlane Laboratories, Hornby, Ont., Canada), which recognizes murine natural killer (NK) cells and depletes NK activity *in vivo* (Kasai *et al.*, 1980; Shpitz *et al.*, 1994).

*Corresponding author. Tel.: +1-530-752-2884. Fax: +1-530-752-4669. E-mail: megershwin@ucdavis.edu

ISSN 1740-2522 print/ISSN 1740-2530 online © 2003 Taylor & Francis Ltd
DOI: 10.1080/10446670310001642122

A suspension of $2 \times 10^7$ healthy control human PBMC or $4–10 \times 10^6$ B cells purified by anti-human CD19 microbeads (Miltenyi Biotec, Bergisch Gladbach, Germany) obtained following leukophoresis, in 50 μl of phosphate-buffered saline (PBS), was directly injected into the spleen under general anesthesia in groups of at least 30 mice. After the injection, physiological salt solution was frequently injected into the peritoneum to correct for dehydration. Twenty microliters of anti-asialo GM1 antibody was injected i.p. into each mouse every 5 days. To prevent infection, mice were maintained in microisolator cages and provided drinking water supplemented with antibiotics. Three mice were examined weekly. Single cell suspensions of splenocytes were prepared by slicing cells from spleen using the end of slideglass and washed with 0.5% bovine serum albumin (BSA) in PBS (washing buffer). The cells were counted in a hemocytometer and viability of cells determined using trypan blue exclusion.

### Flow Cytometric Analysis

PBMC or splenocytes were resuspended in staining buffer (0.5% BSA, 0.05% sodium azide in PBS) and 25 μl aliquots dispensed into individual tubes. The cells were first pre-incubated with anti-human FcR blocking reagent (Miltenyi Biotec) and anti-mouse Fc receptor antibody (eBioscience, San Diego, CA, USA). Cells were stained with different combinations of the following antibodies for 15 min at 4°C: FITC conjugated anti-human CD45, PE conjugated anti-human CD138, PE-Cy5 conjugated anti-human CD38, APC conjugated anti-human CD19 or CD27 (eBioscience), and Cy-Chrome conjugated anti-human IgM (BD Biosciences, San Diego, CA, USA). The cells were then washed with washing buffer, resuspended with 0.05% EDTA in washing buffer and analyzed with a FACSCalibur (BD Biosciences) flow cytometer. The acquired data were analyzed with CELLQUEST software (BD Biosciences).

### Determination of Human Ig Levels

Serum samples were collected from three mice every week and the concentration of human IgG and IgM determined utilizing an Immuno-Tek human IgM and IgG EIA Kit (ZeptoMetrix Corporation, Buffalo, NY, USA). Known positive and negative control samples were included throughout.

### RESULTS

### Transfer of Human PBMC into NOD/SCID Mouse Spleen

To examine the development and differentiation of human B cells into plasma cells in mouse spleen, we injected $2 \times 10^7$ human PBMC or 4 to $10 \times 10^6$ B cells directly into the spleens of NOD/SCID mice that had been γ-irradiated and depleted of NK cell activity. After the injection, spleens were removed from groups of recipient mice at weekly intervals and examined by flow cytometric analysis. As shown in Fig. 1, the number of viable human mononuclear cells (human $CD45^+$ cells, Fig. 1A) from each spleen remained relatively stable for up to 6 weeks after transplantation (Fig. 1C). By week 7, human cells were no longer detected in the spleen. Human B cells and plasma cells, identified as $CD45^+CD19^+CD138^-$ and $CD45^+CD138^+$ cells respectively (Fig. 1B) (Sanderson *et al.*, 1989), were also enumerated weekly. The number of $CD19^+$ B cells in each spleen remained essentially unchanged for the first 2 weeks after transplantation and declined gradually thereafter. In contrast, $CD138^+$ plasma cells while barely detectable in the PBMC before transplantation (week 0), increased by more than 100-fold by week 3 and remained readily detectable up to week 5 (Fig. 1C). The frequency of plasma cells

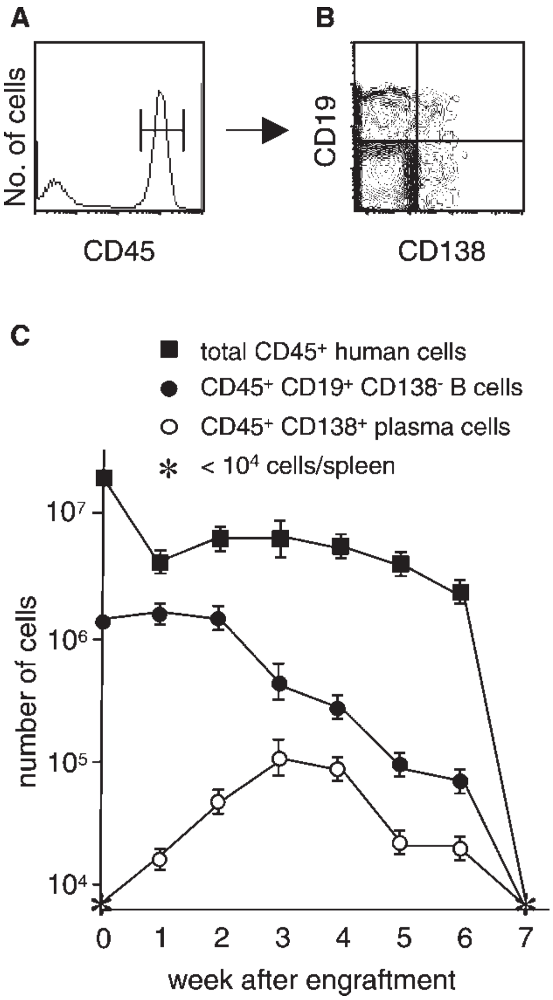

FIGURE 1 Kinetics of human PBMC subsets grafted into NOD/SCID mouse spleen. (A) Human cells are defined as $CD45^+$. (B) Human B cells and plasma cells are defined as $CD45^+CD19^+CD138^-$ and $CD45^+CD138^+$, respectively. (C) The number of total human cells, human B cells and human plasma cells in each spleen at different time points following transplantation.

increased from 0.06% in PBMC before transplantation to 2.1% of the human cells recovered from mouse spleen at week 3. These data indicate that human B cells had differentiated into plasma cells. When purified human B cells were transplanted, no cells were detected even 1 week after transplantation (data not shown).

### Phenotypic Analysis of Human B Cells

Expression of CD27 has been used as a marker to distinguish memory B cells from naïve B cells (Klein *et al.*, 1998; Tangye *et al.*, 1998). We quantified $CD19^+$ B cells that expressed CD27 before transplantation (week 0) and at different time points after transplantation. At week 1 post-transplantation, the number of $CD27^+$ memory B cells per spleen increased while that of $CD27^-$ naïve B cells decreased when compared to those in the transplanted PBMC (week 0) (Fig. 2A), suggesting that differentiation of naïve B cells into memory B cells occurred during the first week after transplantation. The frequency of $CD27^+$ memory B cells in the total $CD19^+$ B cell population increased steadily while the number of both memory and naïve B cells declined after week 1 (Fig. 2B), suggesting that memory B cells have a longer life span than naïve B cells in the mouse spleen. Alternatively, this may indicate that some naïve B cells continued to differentiate into memory B cells during this time period.

We also examined the expression of sIgM by B cells. At all time points examined, both $IgM^+$ and $IgM^-$ memory and naïve B cells were detected. The overall frequency of $IgM^+$ B cells declined from 68% at week 0 to 27% at week 5 after transplantation (Fig. 2C). Of note, most of this change is attributed to the decrease of naïve B cells ($CD27^-$) that expressed IgM. In PBMC before transplantation, 50% of B cells are $CD27^-IgM^+$; while 5 weeks after transplantation, 69% of B cells are $CD27^+IgM^-$ (Fig. 2D), again suggesting that $IgM^-$ memory B cells have a longer life span.

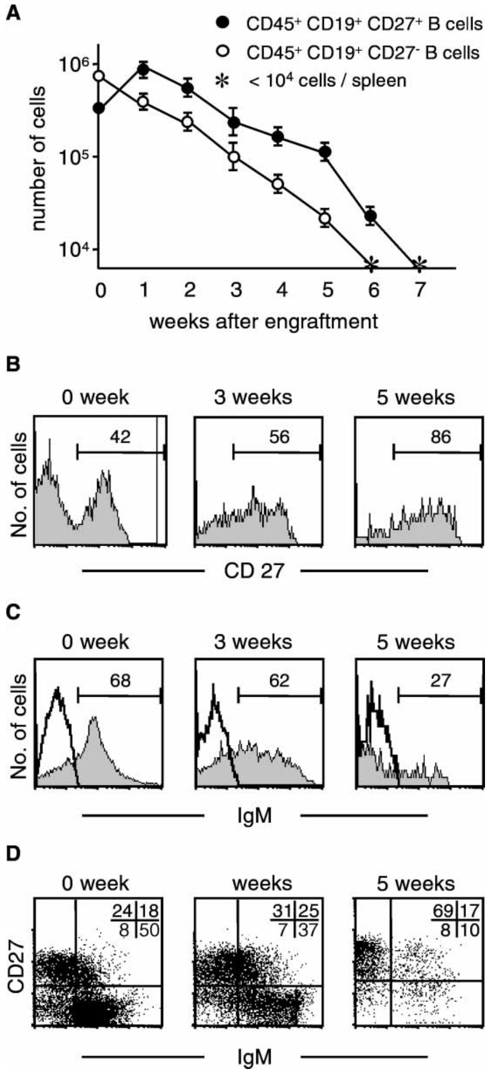

FIGURE 2 Kinetics and phenotype of engrafted human B cells. Human naïve and memory B cells are defined as $CD45^+CD19^+CD27^-$ and $CD45^+CD19^+CD27^+$, respectively. (A) The number of human naïve and memory B cells in each spleen at different time points following transplantation. (B) Kinetics of memory B cell frequency; the number in the histogram is the percentage of $CD27^+$ cells in the $CD45^+CD19^+$ B cell population. (C) Kinetics of surface $IgM^+$ B cell frequency; the number in the histogram is the percentage of surface $IgM^+$ cells in the $CD45^+CD19^+$ B cell population. The unfilled graph indicates the histogram of cells with isotype control antibody. (D) Transition of surface $IgM^+$ cell frequency in naïve and memory B cells. Cells are gated on the $CD45^+CD19^+$ B cell populations. The numbers in the dot plots are percentage of cells in each quadrant.

### Phenotypic Analysis of Human Plasma Cells

We analyzed the expression of the cell surface markers CD19, CD38 and IgM on $CD138^+$ plasma cells recovered from mouse spleen. As shown in Fig. 3A, plasma cells were found in both $CD19^+$ and $CD19^-$ compartments at all time points examined (Fig. 3A). The majority of plasma cells expressed CD38, however, 13% of plasma cells in the CD19 negative population were $CD38^-$ at week 5 (Fig. 3B). Changes in the frequency of $IgM^+$ plasma cells were within the $CD19^+$ and $CD19^-$ populations. Between week 3 to week 5, it declined from 39 to 10% in $CD19^+$ population, but remained unchanged in the $CD19^-$ population (Fig. 3C).

### Detection of Human Immunoglobulin in Mouse Serum

As shown in Fig. 4, the concentration of both human IgG and IgM in mouse serum increased significantly after transplantation. The peak concentration correlated with the peak of plasma cells, indicating that the human plasma cells generated in the mouse spleen are functional. The concentration of IgM started to decline at week 5 and became undetectable at week 7, while that of IgG remained little changed until week 6. By week 7 a significant level of IgG can still be detected, suggesting that the IgG-producing plasma cells have a longer life-span than IgM-producing cells.

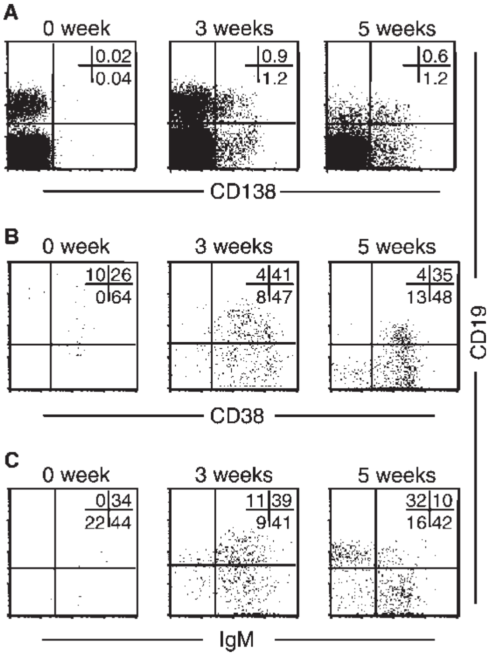

FIGURE 3 Phenotype of human plasma cells recovered from mouse spleen. Human plasma cells are defined as $CD45^{+}CD138^{+}$. (A) Frequency of $CD19^{+}$ and $CD19^{-}$ plasma cells; the numbers in the dot plots are the percentage of $CD19^{+}$ and $CD19^{-}$ plasma cells in the recovered $CD45^{+}$ human cells. (B) Expression of CD38 and CD19 by $CD138^{+}$ plasma cells. Cells are gated on $CD45^{+}CD138^{+}$ plasma cell population; the numbers in the dot plots are percentage of cells in each quadrant. (C) Expression of cell surface IgM and CD19 on $CD138^{+}$ plasma cells; the numbers in the dot plots are the percentage of cells in each quadrant.

### DISCUSSION

We studied herein the differentiation of human B cells using a SCID-Hu mouse model. Human PBMC were transplanted into spleens of NOD/SCID mice following irradiation and depletion of NK cell activity. Human cells were found to survive in the mouse spleen for up to 6 weeks. The number of plasma cells greatly increased, with high circulating levels of both human IgG and IgM. Our results indicate that human B cells can differentiate into mature plasma cells in the murine spleen. To our knowledge this is the first observation of newly differentiated plasma cells in SCID-Hu mice using the direct intrasplenic injection method.

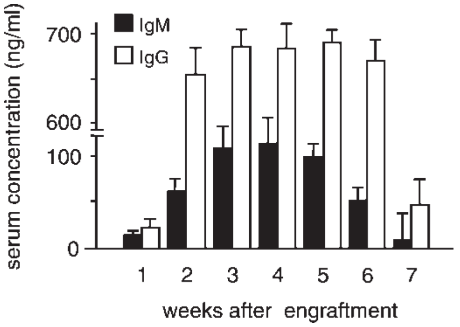

FIGURE 4 Serum concentration of human immunoglobulins in mice receiving human PBMC.

Spleen is known to be a major site for B cell differentiation and plasma cell maturation, a process that has not been fully characterized. A recent study found that when human PBMC were engrafted into the spleen of irradiated NOD/SCID mice, B cells became activated and differentiated into plasmacytoid cells (Depraetere *et al.*, 2001). However, the engrafted B cells were followed for a relatively short time period, because almost all mice died of graft-vs-host disease (GVHD) within 3–4 weeks after transplantation. For this reason, terminally differentiated plasma cells have not been characterized in detail (Depraetere *et al.*, 2001). Successful transplantation of human cells into mice requires both irradiation and depletion of NK cell activity in recipient mice (Shultz *et al.*, 1995). Irradiation of the mice may augment the capacity of murine spleen for the differentiation of transplanted human cells. However, high dose irradiation may also cause severe GVHD, resulting in early death of mice that limits the time span for following transplanted human cells. In this study, we irradiated the mice with a lower dose than that previously reported (2 Gy vs. 3 Gy) (Depraetere *et al.*, 2001). Our procedure resulted in a significantly improved animal survival of 9 weeks after transplantation (data not shown), without compromising the capability of irradiated mouse spleen to support the differentiation of engrafted B cells.

Antigen stimulation followed by T cell help initiates naïve B cells differentiation into memory B cells and plasma cells. For B cell differentiation to occur, activated T cell help including CD40/CD40L interactions are required. This requirement has also been shown in SCID-Hu mice. In addition, interaction of tumor-necrosis factor (TNF) family ligands and their receptors play an important role in the splenic environment for B cell differentiation (Fu and Chaplin, 1999). Recently, a member of TNF family, BLyS (B lymphocyte stimulation, also named BAFF or TALL-1) has been found to express on T cells, dendritic cells, monocytes and macrophages. We postulate that injection of human PBMC into the spleen of NOD/SCID mouse leads to dysregulation of BLyS and/or its receptor, resulting in the differentiation of B cells and generation of plasma cells. Since transplantation of purified human B cells into the mouse spleen did not lead to similar results, we believe that other non-B cell factors are necessary in the differentiation and maturation of B cells.

Naïve B cells are generated in the bone marrow. From there they migrate into spleen, some stay at the marginal zone while others localize to the follicle. In the marginal zone, naïve B cells are activated by antigen. The activated B cells form foci in the extrafollicular region and differentiate into plasma cells that produce low-affinity IgM as a primary antibody response (Calame, 2001). The IgM-producing plasma cells die by apoptosis after a short life span (Smith *et al.*, 1996; Dal Porto *et al.*, 1998). On the other hand, if the follicle occurs within the germinal center, they become memory B cells and switch isotype into high affinity IgG-producing plasma cells facilitated by CD40/CD40L interactions (Arpin *et al.*, 1995). These cells home to the bone marrow and their life span is long

(Slifka *et al.*, 1998). For germinal center B cells, the expression of CD38 inhibits apoptosis (Zupo *et al.*, 1994), suggesting that the $CD38^+$ population is long-lived. It has been reported that plasma cells which differentiate in the spleen germinal center were $CD19^+CD138^+$ (Angelin-Duclos *et al.*, 2000), a phenotype called early plasma cells (Jego *et al.*, 1999).

Our finding is in agreement with the thesis that naïve B cells are activated and differentiate into memory B cells within the spleen. During the first week after engraftment, the number of $CD27^-$ naïve B cells declined while that of $CD27^+$ memory B cells increased (Fig. 2A). The greatly increased number of plasma cells after transplantation as well as the concurrent detection of high levels of human IgG and IgM in the mouse serum indicates that human B cells have differentiated into functional plasma cells. At week 3 after transplantation, most plasma cells express IgM (Fig. 3C), suggesting these are the short-life IgM-producing plasma cells which differentiate in the extrafollicular region (Smith *et al.*, 1996; Dal Porto *et al.*, 1998). By week 5, most of the plasma cells were $CD38^+IgM^-$, suggesting that they are IgG-producing long-life plasma cells (Arpin *et al.*, 1995; Slifka *et al.*, 1998).

In this work we used CD138 as the marker to define plasma cells because it has been reported to be a more specific marker for plasma cells than CD38, as the latter is also expressed by activated B cells or plasmablasts (Sanderson *et al.*, 1989; Wijdenes *et al.*, 1996; Jego *et al.*, 1999; Deaglio *et al.*, 2001). With the use of this marker, we found that in addition to $CD19^+CD138^+$ plasma cells as previously described (Jego *et al.*, 1999), $CD19^-CD138^+$ plasma cells were also detected (Fig. 3A). It is known that when $CD19^+$ plasma cells migrate from the peripheral blood to bone marrow, CD19 expression is down-regulated during the process of maturation (Medina *et al.*, 2002). Our observation suggests that the downregulation of CD19 expression may also occur in the spleen. However, the origin and function of the $CD19^-$ plasma cells we identified need to be further characterized.

Thus we have established a SCID-Hu mouse model that appears to be physiologically capable to allow the differentiation process of human B cells into mature and functional plasma cells. A traceable population of $CD138^+$ plasma cells can be followed for up to 6 weeks after transplantation of human PBMC into the mouse spleen. Differentiation of B cells and generation of plasma cells plays important roles in the regulation of normal and abnormal B cell immunity. This system will be useful for studying the generation of protective antibodies against infection, as well as the generation of antibodies involved in autoimmune diseases.

***Acknowledgements***

We thank Tomoyuki Okada, Gregory Nalbandian and Eiko Kikuchi for their kind assistance. Supported by National Institutes of Health Grant CA 20408.

***References***

Angelin-Duclos, C., Cattoretti, G., Lin, K.I. and Calame, K. (2000) "Commitment of B lymphocytes to a plasma cell fate is associated with Blimp-1 expression *in vivo*", *J. Immunol.* **165**, 5462–5471.

Arpin, C., Dechanet, J., van Kooten, C., *et al.* (1995) "Generation of memory B cells and plasma cells *in vitro*", *Science* **268**, 720–722.

Calame, K.L. (2001) "Plasma cells: finding new light at the end of B cell development", *Nat. Immunol.* **2**, 1103–1108.

Dal Porto, J.M., Haberman, A.M., Shlomchik, M.J. and Kelsoe, G. (1998) "Antigen drives very low affinity B cells to become plasmacytes and enter germinal centers", *J. Immunol.* **161**, 5373–5381.

Deaglio, S., Mehta, K. and Malavasi, F. (2001) "Human CD38: a (r)evolutionary story of enzymes and receptors", *Leuk. Res.* **25**, 1–12.

Depraetere, S., Verhoye, L., Leclercq, G. and Leroux-Roels, G. (2001) "Human B cell growth and differentiation in the spleen of immunodeficient mice", *J. Immunol.* **166**, 2929–2936.

Fu, Y.X. and Chaplin, D.D. (1999) "Development and maturation of secondary lymphoid tissues", *Annu. Rev. Immunol.* **17**, 399–433.

Hogan, C.J., Shpall, E.J., McNulty, O., *et al.* (1997) "Engraftment and development of human CD34(+)-enriched cells from umbilical cord blood in NOD/LtSz-scid/scid mice", *Blood* **90**, 85–96.

Jego, G., Robillard, N., Puthier, D., *et al.* (1999) "Reactive plasmacytoses are expansions of plasmablasts retaining the capacity to differentiate into plasma cells", *Blood* **94**, 701–712.

Kasai, M., Iwamori, M., Nagai, Y., Okumura, K. and Tada, T. (1980) "A glycolipid on the surface of mouse natural killer cells", *Eur. J. Immunol.* **10**, 175–180.

Klein, U., Rajewsky, K. and Kuppers, R. (1998) "Human immunoglobulin (Ig)M+IgD+ peripheral blood B cells expressing the CD27 cell surface antigen carry somatically mutated variable region genes: CD27 as a general marker for somatically mutated (memory) B cells", *J. Exp. Med.* **188**, 1679–1689.

McCune, J.M., Namikawa, R., Kaneshima, H., Shultz, L.D., Lieberman, M. and Weissman, I.L. (1988) "The SCID-hu mouse: murine model for the analysis of human hematolymphoid differentiation and function", *Science* **241**, 1632–1639.

Medina, F., Segundo, C., Campos-Caro, A., Gonzalez-Garcia, I. and Brieva, J.A. (2002) "The heterogeneity shown by human plasma cells from tonsil, blood, and bone marrow reveals graded stages of increasing maturity, but local profiles of adhesion molecule expression", *Blood* **99**, 2154–2161.

Mosier, D.E., Gulizia, R.J., Baird, S.M. and Wilson, D.B. (1988) "Transfer of a functional human immune system to mice with severe combined immunodeficiency", *Nature* **335**, 256–259.

Novelli, E.M., Ramirez, M., Leung, W. and Civin, C.I. (1999) "Human hematopoietic stem/progenitor cells generate CD5+ B lymphoid cells in NOD/SCID mice", *Stem Cells* **17**, 242–252.

Pflumio, F., Izac, B., Katz, A., Shultz, L.D., Vainchenker, W. and Coulombel, L. (1996) "Phenotype and function of human hematopoietic cells engrafting immune-deficient CB17-severe combined immunodeficiency mice and nonobese diabetic-severe combined immunodeficiency mice after transplantation of human cord blood mononuclear cells", *Blood* **88**, 3731–3740.

Rossi, M.I., Medina, K.L., Garrett, K., *et al.* (2001) "Relatively normal human lymphopoiesis but rapid turnover of newly formed B cells in transplanted nonobese diabetic/SCID mice", *J. Immunol.* **167**, 3033–3042.

Sanderson, R.D., Lalor, P. and Bernfield, M.B. (1989) "lymphocytes express and lose syndecan at specific stages of differentiation", *Cell Regul.* **1**, 27–35.

Shpitz, B., Chambers, C.A., Singhal, A.B., *et al.* (1994) "High level functional engraftment of severe combined immunodeficient mice with human peripheral blood lymphocytes following pretreatment with radiation and anti-asialo GM1", *J. Immunol. Methods* **169**, 1–15.

Shultz, L.D., Schweitzer, P.A., Christianson, S.W., *et al.* (1995) "Multiple defects in innate and adaptive immunologic function in NOD/LtSz-scid mice", *J. Immunol.* **154**, 180–191.

Slifka, M.K., Antia, R., Whitmire, J.K. and Ahmed, R. (1998) "Humoral immunity due to long-lived plasma cells", *Immunity* **8**, 363–372.

Smith, K.G., Hewitson, T.D., Nossal, G.J. and Tarlinton, D.M. (1996) "The phenotype and fate of the antibody-forming cells of the splenic foci", *Eur. J. Immunol.* **26**, 444–448.

Tangye, S.G., Liu, Y.J., Aversa, G., Phillips, J.H. and de Vries, J.E. (1998) "Identification of functional human splenic memory B cells by expression of CD148 and CD27", *J. Exp. Med.* **188**, 1691–1703.

Wijdenes, J., Vooijs, W.C., Clement, C., *et al.* (1996) "A plasmocyte selective monoclonal antibody (B-B4) recognizes syndecan-1", *Br. J. Haematol.* **94**, 318–323.

Zupo, S., Rugari, E., Dono, M., Taborelli, G., Malavasi, F. and Ferrarini, M. (1994) "CD38 signaling by agonistic monoclonal antibody prevents apoptosis of human germinal center B cells", *Eur. J. Immunol.* **24**, 1218–1222.

